# Tumor-Specific Peptide, Selected from a Phage Peptide Library, Enhances Antitumor Activity of Lactaptin

**DOI:** 10.1371/journal.pone.0160980

**Published:** 2016-08-11

**Authors:** Anna A. Nemudraya, Anna A. Makartsova, Alexandr S. Fomin, Anna A. Nushtaeva, Olga A. Koval, Vladimir A. Richter, Elena V. Kuligina

**Affiliations:** 1 Institute of Chemical Biology and Fundamental Medicine SB RAS, Novosibirsk, Russia; 2 Novosibirsk State University, Novosibirsk, Russia; Karolinska Institutet, SWEDEN

## Abstract

A recombinant analogue of lactaptin (RL2), a new potential anticancer molecule, induces apoptosis in cultured tumor cells. The tumor suppression efficacy of RL2 was shown against mouse hepatoma-1 cells and MDA-MB-231 human breast adenocarcinoma cells. The RL2-based therapeutic drug lactaptin is distributed evenly throughout the organism, which reduces its antitumor efficacy. In the current study, we obtained a genetic construct that allows production of the recombinant fusion protein T3-RL2, consisting of RL2 and T3 peptide (YTYDPWLIFPAN), in *E*. *coli* cells. T3 peptide was selected from a phage peptide library as a result of two screenings: *in vitro* using MDA-MB-231 cell culture and *in vivo* using a mouse xenograft model of breast cancer MDA-MB-231. It was shown that the displayed peptide T3 provides binding and internalization of phage particles by MDA-MB-231 cells and their specific accumulation in MDA-MB-231 tumor tissue. In addition, based on the nucleotide sequences coding RL2 and the known tumor-targeting peptide iRGD, we obtained genetic constructs that provide synthesis of fusion proteins RL2-iRGD and RL-iRGD-His. We studied the cytotoxic activity of fusion proteins T3-RL2, RL2-iRGD and RL-iRGD-His *in vitro* using MDA-MB-231 and MCF-7 human adenocarcinoma cells. The *in vitro* results showed that the fusion proteins inhibit proliferation of both cell cultures, and their cytotoxic activity is higher than that of RL2. *In vivo* experiments on the study of the antitumor efficacy of the obtained fusion proteins demonstrated that T3-RL2 protein significantly inhibits MDA-MB-231 tumor growth in a xenograft model compared with RL2, while the antitumor effect of RL2-iRGD and RL-iRGD-His proteins is comparable to the effect of RL2.

## Introduction

Lactaptin (~8.6 kDa), a proteolytic fragment of human kappa-casein (residues 57–134), has been previously found in human breast milk. Lactaptin was capable of reducing cell viability and inducing apoptosis in cultured tumor cells [1]. A series of recombinant analogues of lactaptin were constructed, but only one of them (RL2) effectively induced cell death in various human cancer cells (breast adenocarcinomas MCF-7 and MDA-MB-231, lung carcinoma A549, larynx epidermal carcinoma HEp-2) and mouse cancer cells (Lewis lung carcinoma and HA1 hepatoma) while having no effect on the viability of non-malignant MSC cells [2]. The tumor suppression efficacy of RL2 *in vivo* was shown against mouse hepatoma-1 as well as human adenocarcinoma MDA-MB-231 cells grafted onto severe combined immunodeficiency (SCID) mice [3, 4]. The preclinical trials of the RL2-based therapeutic drug lactaptin have been successful, and the safety and antitumor efficacy of this drug have been demonstrated. However, lactaptin, as most protein-based therapeutic drugs, is distributed evenly throughout the organism, which reduces its antitumor efficacy [5]. Further improvements for the enhancement of lactaptin efficacy by rational modifications are required.

Currently, various modifications to obtain targeting properties are considered as one of the most promising ways to improve the therapeutic effect of antitumor drugs. In particular, direct conjugation to a tumor-specific peptide could increase the local concentration of the drug in tumor tissue.

An effective method of obtaining tumor-specific peptides is screening of phage peptide libraries, which is carried out *in vitro* using cancer cell cultures and *in vivo* using animal models [6]. A successful example of such screening is the iRGD peptide (CRGDK/RGPDC), which combines two amino acid motifs (RGD motif and R/KXXR/K motif) and two properties: the ability to specifically bind to cancer cells and the ability to internalize into cells and increase the permeability of blood vessels and tumor parenchyma. The complete mechanism of iRGD action was described by Sugahara et al. [7]. The peptide iRGD has been shown to enhance the antitumor efficacy of agents upon their conjugation/fusion while acting as a delivery agent [8, 9].

We hypothesized that the conjugation of a short peptide specific to tumor tissue to RL2 could enhance its antitumor efficacy *in vivo*. In this study, we performed biopanning of a phage peptide library *in vitro* on human breast cancer MDA-MB-231 cells and *in vivo* on a human breast cancer MDA-MB-231 xenograft model. We selected a phage clone displaying T3 peptide that had the greatest specificity for cancer cells and tumor tissue. Based on the nucleotide sequences coding the selected peptide (T3) and iRGD peptide, recombinant plasmids were constructed that provided synthesis of fusion proteins T3-RL2, RL-iRGD-His and RL2-iRGD consisting of tumor-specific peptides and cytotoxic protein RL2. A comparative assessment of the cytotoxic activity of the fusion proteins was conducted *in vitro* on human cancer cells and their antitumor efficacy was investigated *in vivo* in tumor models.

## Materials and Methods

### Cell cultures

Cancer cell lines MDA-MB-231 and MCF-7 were obtained from the Russian cell culture collection (Russian Branch of the ETCS, St. Petersburg, Russia). MDA-MB-231 cells were cultivated in Leibovitz’s (L15) medium (Sigma) supplemented with 10% FBS, 2 mM L-glutamine, 250 mg/mL amphotericin B and 100 U/mL penicillin/streptomycin. Cells were grown in a humidified 5% CO_2_–air atmosphere at 37°C and were passaged with 0.05% trypsin-EDTA every 3–4 days. MCF-7 cells were cultivated in Iscove’s modified Dulbecco’s medium (IMDM, Sigma) with 10% FBS (Gibco BRL Co., Gaithersburg, MD), 2 mM L-glutamine (Sigma), 250 mg/mL amphotericin B and 100 U/mL penicillin/streptomycin (Gibco BRL Co., Gaithersburg, MD).

Primary culture of non-transformed human breast BN-2 cells (human breast biopsy was provided with informed consent from patients at the Center of New Medical Technologies, Novosibirsk) was cultured in IMDM supplemented with 10 mM L-glutamine, 200 U/mL penicillin, 200 mg/mL streptomycin sulfate and 500 mg/mL amphotericin B in the presence of 10% FBS. The study protocol was approved by the Institute of Molecular Biology and Biophysics SB RAMS Ethics Committee in accordance with the Declaration of Helsinki of 1975.

### Animals

Female SCID hairless outbred (SHO-*Prkdc*^*scid*^*Hr*^*hr*^) mice aged 6–8 weeks were obtained from the SPF vivarium of the Institute of Cytology and Genetics of the Siberian Branch of the Russian Academy of Science (SB RAS) (Novosibirsk, Russia). Mice were housed in individually ventilated cages (Animal Care Systems, Colorado, USA) in groups of one to four animals per cage with *ad libitum* food (ssniff, Soest, Germany) and water. Mice were kept in the same room within a specific pathogen-free animal facility with a regular 14/10 h light/dark cycle (lights on at 02:00 h) at a constant room temperature of 22 ± 2°C and relative humidity of approximately 45 ± 15%.

All animal experiments were carried out in compliance with the protocols and recommendations for the proper use and care of laboratory animals (EEC Directive 86/609/EEC). The protocol was approved by the Committee on the Ethics of Animal Experiments of the Administration of SB RAS (Permit Number: 8–2012).

### *In vitro* biopanning

Before biopanning on cancer cells, the phage peptide library (Ph.D.™-12 Phage Display Peptide Library Kit, New England BioLabs, USA) was depleted on a primary culture of untransformed human breast BN-2 cells. To do this, BN-2 cells were incubated with the phage peptide library (0.3 × 10^11^ PFU/mL) at 37°C for 1 h. The depleted library contained in the supernatant was amplified according to the manufacturer’s protocol and used in biopanning.

Biopanning of the phage peptide library on MDA-MB-231 cancer cells was performed as described previously with some modifications [10].

#### Round I

MDA-MB-231 cells which had reached 70–80% confluence were incubated with a blocking buffer (5% BSA/PBS) at 37°C for 10 min. After blocking, the cells were washed with PBS, and the depleted phage peptide library (0.3 × 10^11^ PFU/mL) in PBS-BSA Ca/Mg buffer (0.1% BSA/PBS, 1 mM CaCl_2_, 10 mM MgCl_2_*6H_2_O) was added. Cells were incubated with the phage peptide library at 4°C for 1 h. Then, cells were washed four times with PBS and incubated with RPMI medium pre-warmed to 37°C for 15 min for receptor internalization. After incubation, cells were washed four times with PBS, and trypsinization was performed in order to eliminate non-internalized phages. After 2–3 min, the cells were washed four times in PBS and lysed with 1 mL of water. The resulting lysate was used for phage amplification for the subsequent rounds of selection.

#### Rounds II–IV

MDA-MB-231 cells that had reached 70–80% confluence were incubated with blocking buffer (5% BSA/PBS) at 37°C for 10 min. After blocking, the cells were washed with PBS, and the phage peptide library (0.3 × 10^11^ PFU/mL) in PBS-BSA Ca/Mg buffer was added for 1 h at 4°C. Cells were washed four times with PBS and incubated with RPMI medium pre-warmed to 37°C for 1 h at 37°C. Then, cells were washed four times with 0.2% PBST (0.2% Tween 20/PBS) pH 5.0, four times with 0.2% PBST pH 7.4, and trypsinization was performed. After 2–3 min cells were washed four times with PBS and lysed in 1 mL of water. After each round of selection, the resulting suspension of bacteriophages was amplified and used for subsequent rounds.

### *In vivo* biopanning

For *in vivo* experiments, a suspension of MDA-MB-231 cells (3.5–4 × 10^7^ cells/mL) in PBS was mixed with Matrigel (BD Bioscience) at a ratio of 1:1, and 0.1 mL of suspension was injected subcutaneously in the back of each mouse. A mouse with a subcutaneously grafted MDA-MB-231 tumor grown to 0.7–0.8 cm in diameter was subjected to 0.5 mL of the phage display peptide library (2 × 10^9^ PFU/mL) (New England BioLabs, USA) diluted in saline via tail vein injection. Phage particles were allowed to circulate for 5 min, after which the mouse was sedated with 2.5% avertin solution at a dose of 0.2 mL per 20 g of mouse body weight, and the heart was perfused with 15 mL of saline in order to remove the unbound phage particles from the bloodstream. The tumor was removed, washed with PBS and homogenized in 1 mL of PBS containing 1 mM of the protease inhibitor phenylmethylsulfonyl fluoride (PMSF). Homogenate was centrifuged at 10,000 *g* for 10 min at room temperature; the supernatant was removed. Pellets were resuspended in 1 mL of blocking buffer and centrifuged at 10,000 *g* for 10 min at room temperature; the supernatant was removed. Pellets were resuspended in 1 mL of liquid *E*. *сoli* ER2738 culture in LB medium (OD_600_ 0.2–0.3) for elution of the bacteriophages bound to the tumor and incubated at 37°C for 30 min. The eluate containing phage particles was centrifuged at 10,000 *g* for 10 min at room temperature. The suspension containing phage particles was amplified. Amplified phage particles were used in further rounds of selection.

### Sequencing of phage DNA

After the third/fourth round of selection, phage particles were titrated to obtain individual phage colonies, which were used for DNA isolation according to the manufacturer’s protocol for the phage display peptide library.

Single-stranded DNA molecules were used for Sanger sequencing (-96 gIII sequencing primer (5`-CCC TCA TAG TTA GCG TAA CG-3`)) (New England BioLabs, USA). The sequencing reaction products were determined using an ABI 310 Genetic Analyzer (Applied Biosystems, USA) at the Genomics Core Facility of SB RAS. Nucleotide sequences of the inserts encoding peptides were analyzed using MEGA 4.0 software.

### *In vitro* phage binding assays

MDA-MB-231, MCF-7 and BN-2 cells were incubated in 24-well culture plates to 70–80% confluence. Cells were washed twice with PBS and incubated with blocking buffer (5% BSA/PBS) at 37°C for 10 min. After blocking, the cells were washed with PBS and incubated with the selected phage (10^9^ PFU/mL) in PBS-BSA Ca/Mg buffer at 4°C for 1 h. Cells were washed four times with PBS and incubated with RPMI medium pre-warmed to 37°C for 1 h at 37°C. Then, cells were washed four times with PBST pH 5.0, then four times with PBST pH 7.4 and four times with PBS. After washes, cells were lysed in 500 μL of water. The resulting lysates containing phages were titered on LB agar medium supplemented with 1 mg/mL X-Gal and 1.25 mg/mL IPTG in order to count individual phage colonies.

### Flow cytometry

MDA-MB-231, MCF-7 and BN-2 cells that had reached 80–90% confluence were detached with 10 mM EDTA/PBS pH 8.0. Cells were washed with PBS and then incubated at 37°C for 2 h in the growth medium corresponding to the cell line. Next, cells were washed with buffer A (1% FCS/PBS) and incubated for 30 min on ice with the bacteriophage clone (10^10^ PFU/mL) in PBS-BSA Ca/Mg buffer. After incubation with bacteriophages, cells were washed with buffer A and incubated with buffer B (10% FBS/PBA) on ice for 15 min. Cells were incubated with mouse Anti-M13 Bacteriophage Coat Protein g8p antibody (RL-ph1) (primary antibody) (ab9223, Abcam, England) diluted in buffer A (1:200) for 30 min on ice. Cells were washed with buffer A and incubated with Alexa Fluor 488 donkey anti-mouse IgG (H+L) (Invitrogen, USA) diluted in buffer A (1:200) for 30 min on ice. Cells were washed with buffer A, resuspended in 500 μL of buffer A and used for analysis on a BD FACS Canto II flow cytometer.

### Immunocytochemistry

MDA-MB-231 cells were incubated on BD Falcon culture slides to 80–90% confluence, washed with PBS twice, and the selected phage clone (2 × 10^10^ PFU/mL) in PBS-BSA Ca/Mg buffer was added. Cells were incubated with the bacteriophage clone for 2 h at either 4°C or 37°C with the following treatment according to the previously described technique with slight modifications [11].

After incubation at 4°C, cells were washed five times with cold buffer H (1% BSA, 0.1% Tween 20/PBS), then incubated with mouse Anti-M13 Bacteriophage Coat Protein g8p antibodies (RL-ph1) diluted in 1% BSA/PBS buffer (1:200) for 45 min at 4°C and washed four times with cold 1% BSA/PBS buffer. After washing, cells were incubated with Alexa Fluor 488 donkey anti-mouse IgG (H+L) antibodies (Abcam, England) diluted in 1% BSA/PBS buffer (1:200) at 4°C for 45 min and washed four times with cold 1% BSA/PBS buffer. Cells were fixed with cold 4% formaldehyde for 10 min and washed twice with PBS.

After incubation at 37°C, cells were washed three times with buffer M (100 mM glycine, 0.5 M NaCl, pH 2.5) at room temperature, fixed with cold 4% formaldehyde for 10 min and washed twice with PBS. Then, 0.2% Triton X100 was added to fixed cells for 10 min, after which the cells were washed twice with PBS. Next, cells were incubated with mouse Anti-M13 Bacteriophage Coat Protein g8p antibodies (RL-ph1) diluted in 1% BSA/PBS buffer (1:200) for 45 min at 4°C and washed four times with cold 1% BSA/PBS buffer. Next, cells were incubated with secondary Alexa Fluor 488 donkey anti-mouse IgG (H+L) antibodies (Abcam, England) diluted in 1% BSA/PBS buffer (1:200) for 45 min at 4°C and washed four times with cold 1% BSA/PBS buffer.

Then the cells were stained with DAPI and analyzed by fluorescence and confocal microscopy.

### Electron microscopy

MDA-MB-231 cells that had reached 80–90% confluence were washed with PBS and detached with 10 mM EDTA/PBS pH 8.0. Then, cells were washed with PBS and incubated in L15 growth medium (10% FBS) at 37°C for 1 h. Cells were further incubated with the bacteriophage clone (2 × 10^10^ PFU/mL) at 37°C for 10 min or 1 h, then precipitated and fixed with 4% paraformaldehyde solution. Fixed precipitates were washed with the medium, postfixed with 1% solution of osmium tetroxide and dehydrated. Next, Epon-Araldite (SPI, USA) was added to the mixture. Ultrathin sections were obtained from the resulting blocks on an EM UC7 ultramicrotome (Leica, Germany). Sections were contrasted with uranyl acetate solution and lead citrate and examined in a JEM 1400 transmission electron microscope (JEOL, Japan) equipped with a Veleta side-input digital camera (Olympus Soft Imaging Solution, Germany).

### Construction of plasmid DNA expressing fusion proteins

Construction of the T3-RL2 fusion protein was carried out based on the nucleotide sequences coding the selected peptide T3 (YTYDPWLIFPAN) and RL2. The RL2 sequence was amplified from pGSDI/RL2 plasmids [2] using primers RL2_F and c1_RL2_R (Table 1). In order to obtain the T3_RL2 fusion DNA sequence encoding peptides T3 and RL2, first, the T3 sequence encoding the selected peptide with the region overlapping with the RL2 sequence was obtained. The T3 sequence was amplified using primers t3_RL2_F and t3_R from the ClonN1.1_target template. Next, the fusion sequence of T3_RL2 DNA was obtained using primers t3_RL2_F and c1_RL2_R. Т3 and RL2 amplicons were used as a template.

**Table 1:** Sequences of oligonucleotides used in the construction of plasmid DNA providing expression of fusion proteins.

Oligonucleotide	Sequence (5`-3`)
RL2_F	AACCAGAAACAACCAGCATGCCATGAGAATGAT
c1_RL2_R	CATCATGGATCCTTAGTGATGGTGATGGTGATGTGATCCGCCGATGGT
t3_RL2_F	CATCATCCATGGGCTATACCTATGATCC
t3_R	GCGAATGGTGGAGGTTCGAACCAGAAACAACCAG
ClonN1.1_target	TATACCTATGATCCGTGGCTGATTTTTCCGGCGAATGGTGGAGGTTCG
RL2_c1_F	CATCATCCATGGGCAACCAGAAACAACCAGCAT
RL2_RGD_R	CATCGGCGGATCACATCACCATCACCATCACGGTGGAGG
His_RGD	GGTGGAGGTTCGTGCCGTGGCGATAAAGGCCCGGATTGC
His_RGD_F	CATCACGGTGGAGGTTCGTGCCGT
His_RGD_R	CATCATGGATCCTTAGCAATCCGGGCC
RGD_His	TGCCGTGGCGATAAAGGCCCGGATTGCGGTGGAGGTTCG
RGD_F	ATCATCCCTACCATCGGCGGATCATGCCGTGGC
RL2_p_R	GATTGCGGTGGAGGTTCGCATCACCATCACCATCACTAAGGATCCATGATG

Construction of the RL2-iRGD fusion protein was performed based on the nucleotide sequences coding the iRGD peptide and RL2. The RL2 sequence was amplified from pGSD/RL2 plasmids using primers RL2_c1_F and RL2_RGD_R. Next, the H_RGD sequence encoding a histidine tag and the iRGD peptide with a region overlapping with the RL sequence was obtained using His_RGD as a template and primers His_RGD_F and His_RGD_R. For obtaining the fusion nucleotide sequence of RL_H_RGD encoding RL2 and iRGD in the same translation frame, primers RL2_c1_F and His_RGD_R were used on the basis of PCR fragments RL and H_RGD.

Construction of the fusion protein RL-iRGD-His was conducted by insertion of the iRGD peptide sequence between the RL2 fragment and the sequence encoding a histidine tag, within the same translation frame. In order to do this, we obtained the RGD_H sequence from the RGD_His template using primers RGD_F and RL2_p_R. Next, the fusion sequence of RL_RGD_H was obtained using primers RL2_c1_F and RL2_p_R. RL and RGD_H amplicons were used as a template.

All obtained amplicons were separated on an agarose gel and isolated from the gel according to the manufacturer’s recommendations (Qiagen, USA). Insertion of DNA fragments T3_RL2/RL_RGD_H/RL_H_RGD encoding fusion proteins into pET-15b plasmids (Novagen, USA) was performed at BamHI and NcoI restriction sites. The recombinant plasmids were cloned into *E*. *coli* TOP10 (Life Technologies).

The sequencing reaction was carried out using a BigDye Terminator Cycle Sequencing Ready Reaction Kit (Applied Biosystems, USA) with further purification of sequencing reaction products using a DyeEx Spin Kit (Qiagen, USA) at the Genomics Core Facility of SB RAS. Primers T7seq_F 5`-TAATACGACTCACTATAGGG-3`and T7seq_R 5`-GCTAGTTATTGCTCAGCGGT-3`were used for the sequencing reaction. Determination of the nucleotide sequences of the purified products of the sequencing reactions was performed on an ABI 310 Genetic Analyzer automatic sequencer (Applied Biosystems, USA). Analysis of the studied nucleotide sequences was performed using MEGA 4.0 computer software.

The recombinant plasmids pET-15b_Т3_RL2, pET-15b_RL2_iRGD and pET-15b_RL_iRGD_His encoding fusion proteins T3-RL2, RL2-iRGD and RL-iRGD-His, respectively, were expressed in *E*. *coli* BL21 (DE3) (Stratagene). The recombinant lactaptin analogues RL2, Т3-RL2, RL2-iRGD and RL-iRGD-His were isolated from *E*. *coli* and purified according to the protocol for RL2 isolation [2].

### МТТ assay

The cytotoxic effects of the recombinant lactaptin analogues RL2, T3-RL2, RL2-iRGD and RL-iRGD-His on human tumor cells were explored using the МТТ assay (3-(4,5-dimethyl-2-thiazolyl)-2,5-diphenyl-2H-tetrazolium bromide, Sigma-Aldrich) according to the protocol described previously [3]. Cells that had reached 30% confluence in a 96-well plate were incubated with protein preparations at various concentrations for 48 h. After incubation, the supernatant was removed, and 200 μL of MTT solution in RPMI 1640 medium (0.5 mg/mL) was added to each well. Cells were incubated at 37°C for 4 h. Formazan crystals were dissolved in 150 μL of DMSO. The optical density of formazan solutions was measured using an Apollo LB912 photometer (Berthold Technologies, Oak Ridge, TN) at a wavelength of 570 nm. Cell viability was determined in relation to the viability of control cells (100%) ± SD in a series of three independent experiments.

### Antitumor activity

A suspension of MDA-MB-231 cells (3.5–4 × 10^7^ cells/mL) in PBS was mixed with Matrigel (BD Bioscience) at a ratio of 1:1, and 0.1 mL of suspension was injected subcutaneously into the back of each mouse. Mice with a subcutaneously grafted MDA-MB-231 tumor that reached 20 ± 10 mm^3^ were divided into experimental and control groups. Recombinant fusion proteins (T3-RL2, RL2-iRGD, RL-iRGD-His) and RL2 were injected intravenously via tail vein in animals three times every second day at a dose of 40 mg/kg in 500 μL of saline. Saline was injected into the control group with the same mode of administration as the studied agents. Seventeen days after the last injection, all mice were sacrificed by cervical dislocation, and the tumors were excised and weighed. Antitumor activity was evaluated in terms of tumor weight.

### Statistical analysis

The data from the cells and mouse experiments were statistically processed using one-way ANOVA. Post hoc testing was completed using Fisher’s least significant differences (LSD); p < 0.05 was considered to be statistically significant. STATISTICA version 10.0 statistical package was used for analysis.

## Results

### *In vitro* biopanning

Before *in vitro* biopanning of the phage peptide library on cancer cells, negative screening on primary human breast BN-2 culture, characterized for the presence of progesterone, estrogen and tyrosine protein kinase receptors (PR+/ERa−/ERb−/HER2−) by RT-PCR (data not shown), was performed. The depleted library was used for four rounds of selection on human breast cancer MDA-MB-231 cells. The sequences of displayed peptides of individual phage clones were identified and analyzed after the third and fourth rounds of selection (Table 2).

**Table 2:** Sequences and frequencies of the peptides displayed by bacteriophages selected after the third (30 clones) and fourth (30 clones) rounds of biopanning on MDA-MB-231 cancer cells.

Clone №	III round		IV round	
	Sequence of the displayed peptide	Frequency (%)	Sequence of the displayed peptide	Frequency (%)
1.1	YTYDPWLIFPAN	26.7	YTYDPWLIFPAN	53.3
1.2	FIPFDPMSMRWE	30	FIPFDPMSMRWE	30
1.3	SLPVYAPALTSR	10	SLPVYAPALTSR	6.7
1.4	AWWDSPRESRVR	6.7	**–**	**–**
1.5	SLPPSLPRFIPW	3.3	SLPPSLPRFIPW	3.3
1.6	SLPLPMFGISYT	3.3	**–**	**–**
1.7	GMDYPYPLFYPA	3.3	**–**	**–**
1.8	GLPSVSLMPRFY	3.3	**–**	**–**
1.9	HLPELMPQLYGA	3.3	**–**	**–**
1.10	RLPSDLFPLTLH	3.3	**–**	**–**
1.11	FLPDLWDDSLLT	3.3	**–**	**–**
1.12	YLPDPGSSFELL	3.3		
1.13	**–**	**–**	SYGSLPAAVFPL	3.3
1.14	**–**	**–**	KLVDTVETVMWA	3.3

Bacteriophages displaying peptides YTYDPWLIFPAN (1.1), FIPFDPMSMRWE (1.2), SLPVYAPALTSR (1.3) and SLPPSLPRFIPW (1.5) were found after both rounds of biopanning on MDA-MB-231 cells. The frequency of the displayed peptide YTYDPWLIFPAN (1.1) increased to 53.3% after the fourth round (26.7% after the third round), while the frequency of peptide FIPFDPMSMRWE (1.2) did not change (30%). It is peculiar that the amino acid motif SLP was found in different peptides that were selected after the third round: SLPVYAPALTSR (1.3), SLPPSLPRFIPW (1.5) and SLPLPMFGISYT (1.6) with a frequency of 16.6%, and in the peptides selected after the fourth round: SLPVYAPALTSR (1.3), SLPPSLPRFIPW (1.5) and SYGSLPAAVFPL (1.13) with a frequency of 13.3%. In order to further investigate the ability to bind with cells *in vitro* and specific accumulation in tumor tissue, phage candidates displaying peptides 1.1–1.3 were chosen.

### *In vitro* binding assays of selected bacteriophage clones

**Fig 1:**
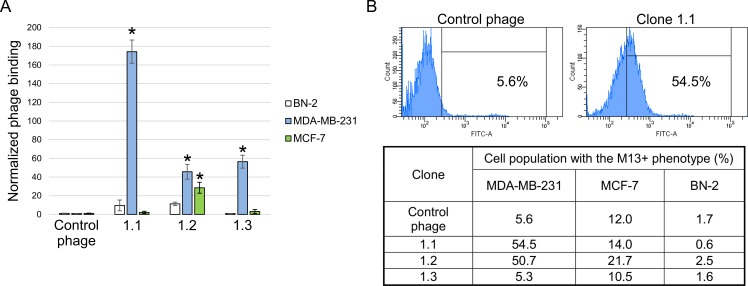
Relative binding of the selected bacteriophage clones to cells. **Displayed peptides 1.1 –**YTYDPWLIFPAN, **1.2 –**FIPFDPMSMRWE **and 1.3 –**SLPVYAPALTSR. (A) MDA-MB-231, MCF-7 and BN-2 cells were incubated with selected phage clones and control bacteriophage. After incubation and a series of washings, the titer of bacteriophages bound to cells was determined. Cells of untransformed human breast BN-2 primary culture were used as a negative control. The value for the binding of the control bacteriophage was used as a normalization value. Data are presented as mean ± SD. Data were statistically analyzed using one-way ANOVA with post hoc Fisher test. A p value < 0.05 was considered to be statistically significant. (B) Flow cytometry of MDA-MB-231, MCF-7 and BN-2 cells incubated with clone 1.1 and control bacteriophage. Flow cytometry was performed using mouse anti-М13 and Alexa Fluor 488 donkey anti-mouse IgG (H+L) antibodies.

Comparative analysis of the binding ability of individual phage clones displaying the selected peptides YTYDPWLIFPAN (1.1), FIPFDPMSMRWE (1.2) and SLPVYAPALTSR (1.3) to cells was performed on human cancer cell lines MDA-MB-231 and MCF-7 and on primary culture of untransformed human breast BN-2 cells (negative control) by measuring the titer of bacteriophages bound to cells by flow cytometry (Fig 1).

According to the data presented in Fig 1, clones 1.1, 1.2 and 1.3 bind more readily to MDA-MB-231 cells compared to the control bacteriophage. Clone 1.1 had the highest binding level; its titer turned out to be about 170 times higher than the negative control titer. In addition, clone 1.2 binds more readily to MCF-7 cells in comparison to the control bacteriophage. It is worth mentioning that clones 1.1–1.3 demonstrated no binding to healthy cells of the primary human breast culture.

Analysis of binding of the selected phage clones to cells by flow cytometry showed an increase to 48.9% in the population of M13-positive cells upon incubation with clone 1.1 and to 45.1% upon incubation with clone 1.2 compared to the control phage (5.6%). In contrast to the results obtained above, no binding of clone 1.3 to MDA-MB-231 cells was revealed. Analysis of the binding of selected clones to MCF-7 cells demonstrated an increase to 9.7% in the population of M13-positive cells upon incubation with clone 1.2 compared to the control phage (12.0%). Analysis of binding to BN-2 cells revealed no significant binding of any of the clones.

### *In vivo* biopanning

Three rounds of phage peptide library biopanning were performed on mice with grafted human tumor MDA-MB-231 in a xenograft model. The sequences of 36 clones of bacteriophages were identified and analyzed after the third round (Table 3).

**Table 3:** Sequences and frequencies of the peptides displayed by bacteriophages selected after the third round of biopanning on mice with grafted human tumor MDA-MB-231 (36 clones).

Clone №	Sequence of the displayed peptide	Frequency (%)
2.1	SLPVYAPALTSR	19.4
2.2	GREPAASLLSHF	16.6
2.3	GTGLVTLPRLTV	13.8
2.4	DSQFNKYSIATV	13.8
2.5	QHYYPIPSDSRS	8.3
2.6	STPPAGPWWSVH	8.3
2.7	SVSIARPMAITK	5.6
2.8	GTDRYPYVLTRG	2.7
2.9	SDNEGVLWSDNT	2.7
2.10	KPGDTAMHYFPP	2.7
2.11	WGTDLTFTTPGT	2.7
2.12	QAYMPSLGLKPV	2.7

The highest frequencies were identified for displayed peptides SLPVYAPALTSR (2.1), GREPAASLLSHF (2.2), GTGLVTLPRLTV (2.3) and DSQFNKYSIATV (2.4) and were equal to 19.4%, 16.6%, 13.8% and 13.8%, respectively. Furthermore, the amino acid sequence of the displayed peptide SLPVYAPALTSR (2.1) corresponds to the amino acid sequence of the displayed peptide of clone 1.3 selected *in vitro* on the human cancer MDA-MB-231 cell line.

### Specific accumulation of selected bacteriophage clones in tumor tissue

**Fig 2:**
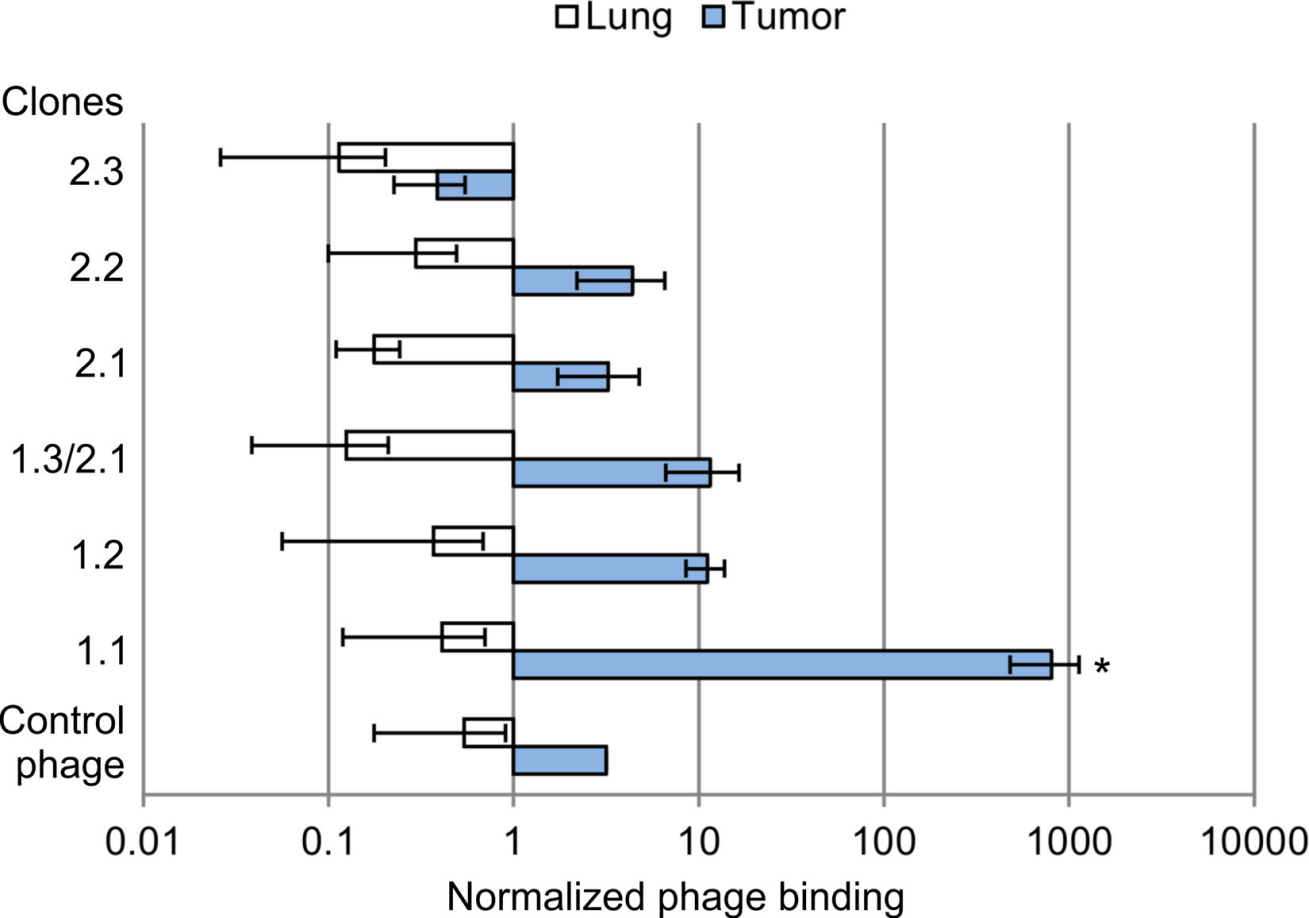
Specific accumulation of the selected phage clones in tumor tissue. Bacteriophage clones displaying peptides 1.1 –YTYDPWLIFPAN, 1.2 –FIPFDPMSMRWE, 1.3 (2.1)–SLPVYAPALTSR, 2.2 –GREPAASLLSHF, 2.3 –GTGLVTLPRLTV, 2.4 –DSQFNKYSIATV and control bacteriophage were injected intravenously into SCID mice with grafted human tumor MDA-MB-231. After 24 h of administration, the bacteriophage titer obtained from the homogenate of tumor tissue, liver and lung was measured. Normalized binding ability to tumor (lung) was calculated as the ratio between the mean titer values (PFU/g) obtained from tumor tissue (lung) and liver. Data were statistically analyzed using one-way ANOVA with post hoc Fisher test. Data are presented as mean ± SEM; a p value < 0.05 was considered to be statistically significant.

The study of specific accumulation of phage clones 1.1–1.3, selected *in vitro*, and clones 2.1–2.4 selected *in vivo* was carried out by determining the bacteriophage titer in tumor tissue of human breast adenocarcinoma MDA-MB-231 grafted into xenograft mice and in control organs (lung and liver) 24 h after intravenous administration of bacteriophage to the tested animals (Fig 2).

**Fig 3:**
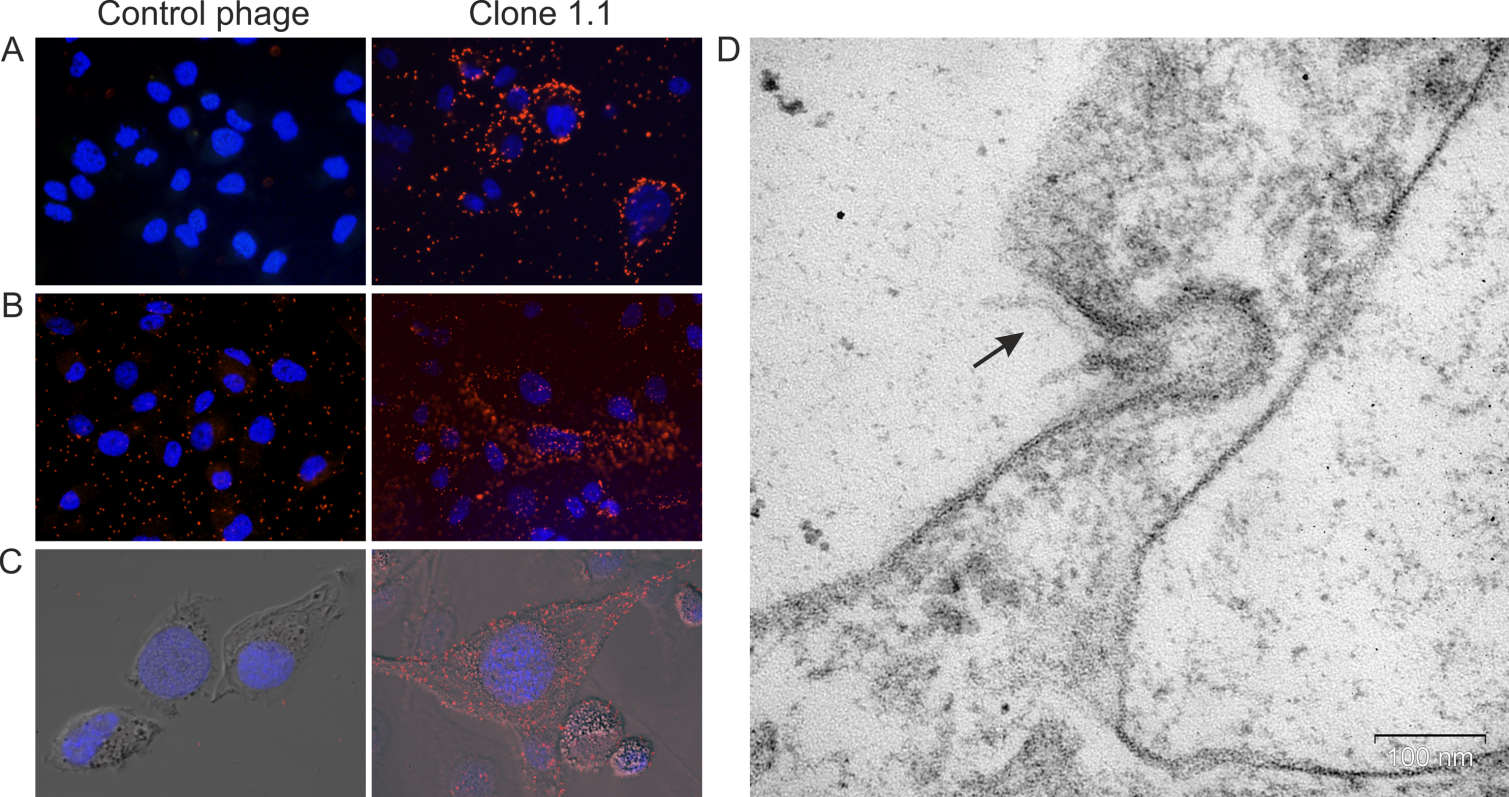
Displayed peptide YTYDPWLIFPAN provided penetration of phage particles into MDA-MB-231 cells. (А) Fluorescence microscopy of MDA-MB-231 cells incubated with phage clone 1.1 and control bacteriophage at 4°C. (B) Fluorescence microscopy and (C) confocal fluorescence microscopy of MDA-MB-231 cells incubated with phage clone 1.1 and control bacteriophage at 37°C and further washes from bacteriophages bound to the surface. Fluorescence microscopy was performed using mouse anti-М13 and Alexa Fluor 488 donkey anti-mouse IgG (H+L) antibodies. For visualization of nuclei, cells were stained with DAPI. (D) Electron microscopy of MDA-MB-231 cell after 1 h of incubation with phage clone 1.1 at 37°C; ultrathin section. The arrow points to a phage particle.

Clone 1.1, which was selected *in vitro*, had the highest binding specificity for MDA-MB-231 tumor tissue. Its titer obtained from tumor tissue was approximately 800-fold higher than the titer obtained from liver and approximately 1900-fold higher than the titer obtained from lung. Other clones and control bacteriophage did not have binding specificity for MDA-MB-231 tumor tissue grafted into SCID mice (Fig 3).

### Interaction of clone 1.1 displaying peptide YTYDPWLIFPAN with MDA-MB-231 cells

Incubation of clone 1.1 displaying peptide YTYDPWLIFPAN with MDA-MB-231 cells was carried out at 4°C in order to confirm binding to the cells and at 37°C in order to evaluate the presence of intracellular phages (Fig 3).

The data in Fig 3 demonstrate that clone 1.1, in contrast to the control bacteriophage, is capable of binding to cell surface structures upon incubation with MDA-MB-231 cells at 4°C. During incubation with MDA-MB-231 cells at 37°C, clone 1.1 remains bound to cells even after a series of washes from bacteriophages, which indicates the ability of the displayed peptide to provide internalization of phage particles. Ultrathin sections of the precipitates of MDA-MB-231 cells after 10 min and 1 h of incubation with phage clone 1.1 showed that phage particles were detected in the intercellular space, in plasmalemma invaginations and bottomed pits, indicating internalization of phage particles by cells via phagocytosis and clathrin-dependent endocytosis.

### Cytotoxic activity of recombinant fusion proteins

Comparative analysis of the effects of RL2 and recombinant fusion proteins T3-RL2, RL2-iRGD and RL-iRGD-His on tumor cell viability was carried out using MDA-MB-231 and MCF-7 cell cultures. Changes in cell viability were assessed using the МТТ assay.

**Fig 4:**
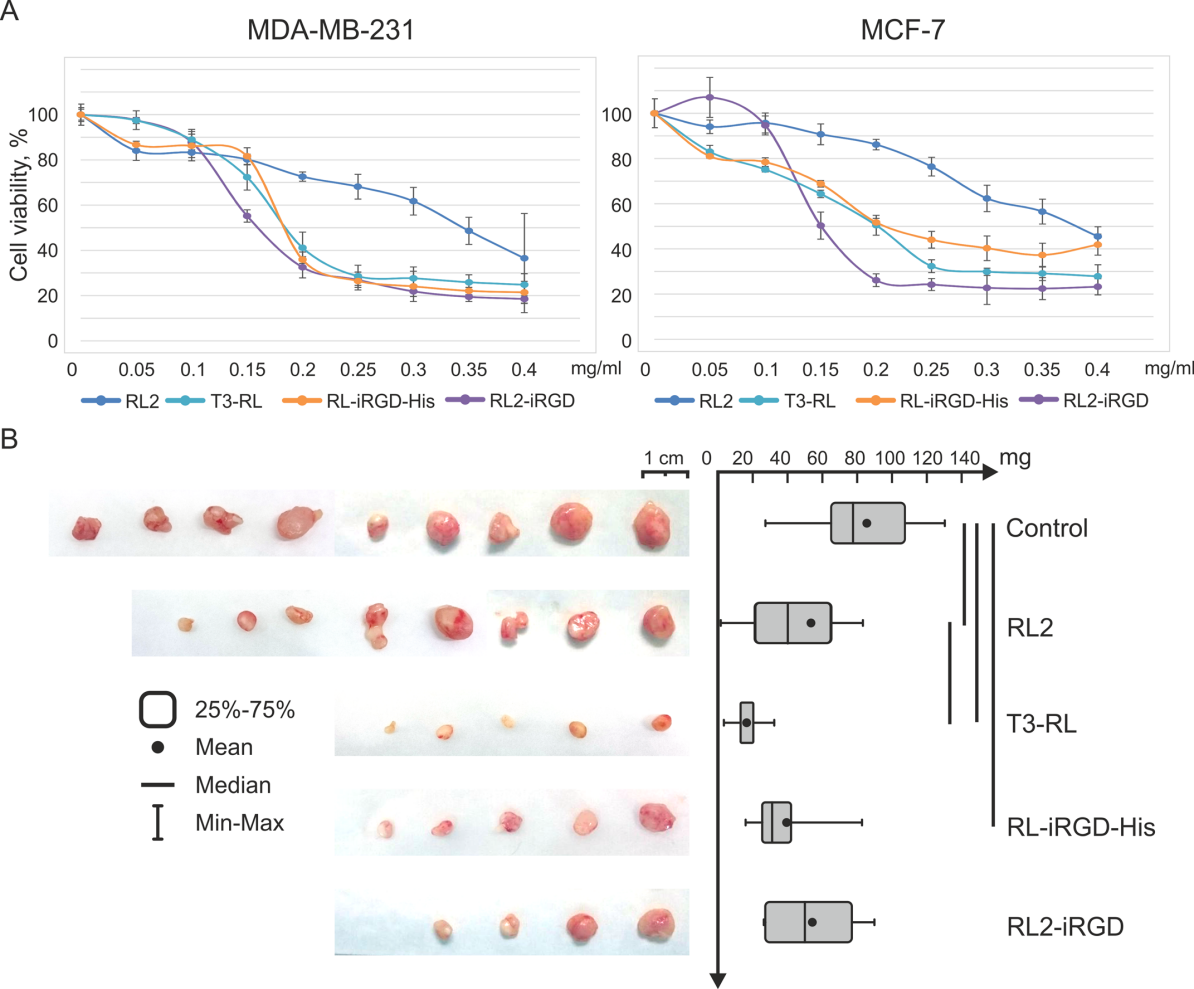
Fusion proteins enhanced cytotoxic outcome and delayed tumor development. (A) MDA-MB-231 and MCF-7 cells were treated with different concentrations of RL2, fusion proteins or saline (control) for 48 h and MTT analysis was performed. Tumor cell viability was determined relative to the viability of the control cells (incubated without the proteins). Data are presented as mean ± SD. (B) SCID mice with subcutaneously grafted MDA-MB-231 tumors, which reached an average (in the group) size of 20 ± 10 mm^3^, were subjected to intravenous injection of fusion proteins (RL2, T3-RL2, RL2-iRGD or RL-iRGD-His) at a dose of 40 mg/kg in saline three times every second day. The control group of mice received saline with the same mode of as administration the proteins. Tumors were excised and weighed on the 17th day after the last injection. Boxes represent the 25th, 50th and 75th percentiles. Squares with lines represent the median. Whiskers represent minimum/maximum. Data were statistically analyzed using one-way ANOVA with post hoc Fisher test; a p value < 0.05 was considered to be statistically significant.

It has been demonstrated that T3-RL2, RL2-iRGD and RL-iRGD-His reduce viability of MDA-MB-231 and MCF-7 cells in a dose-dependent manner in the same way as RL2 (Fig 4). At the same time, the cytotoxic activity of T3-RL2, RL2-iRGD and RL-iRGD-His proteins in relation to cancer cells of both lines is approximately the same (IC_50_ about 0.15–0.20 mg/mL) and significantly exceeds RL2 activity (IC_50_ about 0.35–0.4 mg/mL).

### Antitumor effect of recombinant fusion proteins

In order to compare the antitumor activity of RL2 and recombinant fusion peptides T3-RL2, RL2-iRGD and RL-iRGD-His, a xenograft model of human breast cancer was used. A suspension of MDA-MB-231 cells in Matrigel was subcutaneously injected into 6–8-week-old female SCID mice to form solid tumors. Tumor volumes were monitored every 3 days. When tumors reached roughly 20 mm^3^, recombinant peptides (40 mg/kg) were injected intravenously every 2 days with a total of three injections. The selected dose of recombinant peptides and the treatment regime were based on the therapeutic scheme described previously [3]. There was no animal mortality observed in either experimental or control groups during the experiment. Seventeen days after the last injection, the tumors were excised and weighed (Fig 4).

As shown in Fig 4, tumors were bigger in the control group than in the groups treated with RL2 and fusion proteins. The average weights of the tumors in the control group and the group treated with RL2 were 85.6 mg and 52.8 mg, respectively. Thus, the rate of tumor growth inhibition after RL2 therapy was calculated as 38%. The most pronounced effect of inhibiting tumor growth was observed in the group treated with T3-RL protein, wherein the average weight of the tumor was 16.4 mg, and the rate of tumor growth inhibition was 80%.

## Discussion

The development of novel antitumor drugs depends, on the one hand, on the ability to influence cellular processes and pathways, which differ between cancer and normal cells, and, on the other hand, on the ability of targeting agents to effectively deliver drugs to the tumor. The latter is possible due to multiple genetic and epigenetic changes that occur during oncogenesis and lead to changes in the proteomic profile of the cancer cell [12–14]. Changes in the type, number and location of receptors results in the formation of a unique topographic phenotype of the cell surface. This surface phenotype is the molecular address for the delivery of biomolecules [15].

Dysregulation of one of the key processes of cell activity, apoptosis, is one of the reasons for the development of malignant tumors. Therefore, the development of new inducers and modulators of apoptosis of cancer cells is one of the approaches for the development of modern oncotherapeutic agents [16]. The recombinant analogue of lactaptin, RL2, is an inducer of apoptosis in mouse and human cancer cells. The mechanism leading to cell death after RL2 treatment involves penetration of the peptide into cells, disruption of the inner mitochondrial membrane potential, down-regulation of BCL-2, activation of the executioners caspase-3 and -7 and apoptotic fragmentation of DNA [4]. The RL2-based therapeutic drug lactaptin possesses an inhibitory effect on tumor growth *in vivo* but, as with most protein-based therapeutic drugs, is distributed evenly throughout the organism, which reduces its antitumor efficacy.

In the current study, in order to enhance the antitumor efficacy of lactaptin, we constructed three recombinant fusion proteins T3-RL2, RL2-iRGD and RL-iRGD-His on the basis of the nucleotide sequences coding RL2 and tumor-specific peptides. Т3 peptide (YTYDPWLIFPAN), which we selected from the phage peptide library in this study, and iRGD peptide (CRGDKGPDC), the sequence of which was taken from the literature data, were chosen as tumor-specific peptides.

For the selection of a specific peptide, we conducted biopanning of the phage peptide library against the MDA-MB-231 cell line, as it is one of the most sensitive human cancer cell lines to RL2 *in vitro*. Moreover, RL2 has been shown to inhibit the growth of MDA-MB-231 tumor cells in a mouse xenograft model [4]. MDA-MB-231 is a triple-negative breast cancer (TNBC), defined as a tumor negative for estrogen receptor (ER), progesterone receptor (PR) and human epidermal growth factor receptor 2 (HER2) [17, 18]. The highly aggressive nature of TNBCs, the high probability of metastasis and the absence of targeted drugs led to the active search for actionable molecular targets for the treatment of patients with these tumors [19]. To date, due to various ‘omic’ technologies, an unexpected level of TNBC heterogeneity has been revealed. Subsequently, potentially actionable molecular features in some TNBCs, such as germline BRCA1/2 mutations or ‘BRCAness’, the presence of an androgen receptor and several rare genomic alterations [20, 21], have been revealed. Nevertheless, the search for novel targets still remains a relevant issue.

We chose two biopanning strategies: *in vitro* on MDA-MB-231 cell culture directed to a selection of peptides capable of internalizing a cargo into cells, and *in vivo* on MDA-MB-231 tumors in a xenograft model. We supposed that tumor targeted delivery efficiency may be improved with the ability of peptides to bind to tumor-specific receptors and provide a higher tumor cell internalization rate. Since both of the strategies have their features, we implemented both approaches as part of this study.

*In vitro* biopanning allows selection of peptides specific to cell lines in the absence of knowledge of the particular target they bind to. The advantage of this type of screening is that cell receptors are located in their native context, and this cannot be mimicked using purified membrane proteins. The topography of the cell surface is defined by the expression levels of plasma membrane-bound proteins as well as their arrangement within the membrane. The second advantage can be concluded from the first one: there is no need for prior identification and purification of the cellular target [15, 22]. Furthermore, selection can be modulated by changing washing conditions, for example by selecting only those peptides displayed by bacteriophages that penetrate into cells.

*In vivo* biopanning allows selection of peptides specific to tumor tissue in its natural context. In addition to the characteristics that distinguish cancer stromal cells from normal cells, as well as tumor endothelial cells, there are physical differences between tumor and normal tissues (temperature change, low concentration of oxygen (hypoxia) and reduced pH) that influence selection in the *in vivo* system [23–25].

In our work *in vitro* screening on MDA-MB-231 cell culture and *in vivo* on MDA-MB-231 tumor in a xenograft model resulted in the isolation of different peptides, using the same library. One possible explanation is a limited sample size of the displayed peptides sequences, another one is that two biopanning approaches used in this study are targeting different molecular features [15]. In another work, for example, *in vivo* screening using CL1-5 tumor xenograft model leaded to the isolation the peptide SP5-52 targeting the vasculature, whereas *in vitro* screening against CL1-5 NSCLC cell line resulted in the isolation the peptide SP5-2 penetrating into the tumor with a more diffuse binding pattern [26, 27].

As a result of comparative analysis of the ability of selected clones (phage candidates displaying the peptide) to bind to human breast cancer cells (MDA-MB-231 and MCF-7) and the comparative analysis of their specific accumulation in MDA-MB-231 tumor tissue, clone 1.1 displaying peptide YTYDPWLIFPAN (T3) and selected *in vitro* was chosen. Displayed peptide T3 was shown to provide binding and internalization of phage particles to MDA-MB-231 cells, which means that it can be considered as a delivery agent.

There are two basic approaches for targeted drug delivery. In the first case, the target peptide is conjugated directly to a therapeutic drug. The second approach assumes the delivery agent to be attached to a drug carrier. We chose the first approach based on the fact that conjugation of a short peptide to RL2 should not have a significant effect on the cytotoxic activity of RL2 and its ability to penetrate through the interstitial space of the tumor.

A plasmid that provides synthesis of the fusion protein T3-RL2, wherein T3 located at the N-terminus and RL2 are separated by a glycine spacer, was constructed based on the sequence of the selected peptide T3 and RL2.

There are examples of successful targeted delivery by a conjugation of a short tumor-specific peptides, selected from phage peptides libraries, to a proapoptotic peptide. Work by Cieslewicz M. et al. showed that tail vein injection of an M2pep, selected *in vitro* against tumor associated macrophages (TAMs), fused with a proapoptotic peptide improves survival in tumor-bearing mice by selective reduction of TAMs [28]. Jung H.-K. et al. investigated a hybrid peptide (named Bld-1-KLA) composed of the CSNRDARRC peptide (Bld-1), which binds to bladder tumor cells, and the D-KLAKLAKKLAKLAK (KLA) peptide, which disrupts mitochondrial membrane and induces apoptotic cell death, as a bladder cancer-targeted therapeutic agent. Treatment of tumor-bearing mice with Bld-1-KLA, compared to the control peptide-KLA, resulted in more efficient apoptosis induction of tumor cells and inhibition of tumor growth [29]. In another work Liu W.Q. et al. investigated the ability of PI (sequence, CASPSGALRSC) peptide to deliver a potential therapeutic protein to MDA-MB-231 cells, fusing PI with glutathione-S-transferase (GST, 26 kDa). The authors demonstrated that the transduction of the PI peptide into the cell is partially mediated by macropinocytosis and caveolin-mediated endocytosis. PI was observed to be capable of successfully delivering GST into the cytoplasm of MDA‑MB‑231 cells, and the exogenous protein did not degrade for ≥72 h [30].

The sequence of iRGD peptide (CRGDKGPDC) was used as a target peptide during construction of the recombinant fusion proteins RL2-iRGD and RL-iRGD-His. The peptide iRGD is a tumor-specific tissue-penetrating peptide [7]. The RGD motif in the structure of iRGD peptide recognizes and binds to α_v_β_3/5_, α_5_β_1_ integrin receptors, which are expressed in a large number by tumor endothelial cells and tumor cells [31, 32]. It has been previously shown that MDA-MB-231 cells express α_v_ integrin receptors on their surface in a sufficient amount for specific recognition by the RGD motif [33, 34]. After binding with α_v_ integrins, iRGD is proteolytically processed into CRGDK/R, exposing an active CendR motif at the C-terminus. Interaction of the CendR motif with NRPs initiates an active bulk transport system through the tumor tissue, allowing drugs conjugated to iRGD and even free drugs co-administered with iRGD to extravasate and spread within the tumor tissue [35]. Recently, linear iRGD conjugated to the C-terminus of recombinant proteins has been used to carry drugs into tumor tissues [36, 37].

The peptide iRGD is located at the C-terminus of the RL2-iRGD fusion protein. Fusion protein RL-iRGD-His is designed so that the sequence of the iRGD peptide separates the sequence of the histidine tag from the fragment encoding lactaptin. Theoretically, interaction of the iRGD peptide in the composition of the RL-iRGD-His fusion protein with integrin receptors can lead to cleavage of the protein by proteases, which results in detachment of the histidine tag and release of the C-terminus of the CRGDK motif and binding with NRPs.

The MTT test of the obtained recombinant fusion proteins using human cancer lines MDA-MB-231 and MCF-7 demonstrated that they decrease the viability of both cell lines in a dose-dependent manner and share a similar cytotoxic activity. At the same time, the fusion proteins have higher cytotoxic activity than RL2. Differences in the antitumor effect are more pronounced between proteins. The recombinant fusion protein T3_RL2 turned out to significantly inhibit growth of MDA-MB-231 tumors in a xenograft model (80% decrease), while the antitumor potential of fusion proteins RL2-iRGD and RL-iRGD-His did not differ from that of RL2. It is possible that conjugation of the tumor-specific peptide to the С-terminus of RL2 led to conformationally constrained interaction between the iRGD peptide and the target.

In conclusion, conjugation of a specific peptide YTYDPWLIFPAN to the N-terminus of RL2 via a glycine spacer significantly enhanced its antitumor potential. Thus, the direct conjugation of RL2 with a specific peptide can be considered as a prospective approach of enhancing its antitumor activity.
